# Thoracic Re-irradiation With Definitive External Beam Radiation Therapy Using Intensity Modulated Radiation Therapy: A Case Report

**DOI:** 10.7759/cureus.71540

**Published:** 2024-10-15

**Authors:** Unta Yamamori, Yukihisa Tamaki, Masafumi Uno, Atsushi Ue, Yoko Sonoyama

**Affiliations:** 1 Radiation Oncology, Shimane University Faculty of Medicine, Izumo, JPN

**Keywords:** intensity modulated radiation therapythoracic, lung cancer, radiation therapy, re-irradiation, tolerated dose

## Abstract

Thoracic re-irradiation has a high risk of severe adverse events, and re-irradiation with curative intent has rarely been performed. However, in recent years, with the introduction of intensity-modulated radiation therapy (IMRT) and stereotactic body radiation therapy, it has become possible to deliver high doses to the target lesions while minimizing the doses to surrounding tissues. The patient in this case had a history of definitive radiation therapy for esophageal cancer. The patient developed new lung cancer, which was treated by re-irradiation. We created a radiation treatment plan using IMRT. This allowed us to reduce the dose to organs at risk and deliver a higher dose to the cancer, increasing the potential for cure. The patient has not experienced any severe late adverse events as of three years and six months after treatment. Additionally, the treatment has been sufficiently effective, and the patient remains recurrence-free. To confirm the feasibility of the IMRT plan, we also created a radiation treatment plan using three-dimensional conformal radiation therapy (3D-CRT) and compared it with the IMRT plan. Compared with 3D-CRT, the IMRT plan was able to reduce the dose to organs at risk and meet the dose constraints indicated in multiple studies. The possibility of adverse events such as bronchial hemorrhage, esophageal hemorrhage, bronchial fistula, radiation pneumonitis, esophageal fistula, and pericarditis was significantly reduced.

## Introduction

Advances in chemotherapy and radiation therapy in recent years have improved the overall survival rates of lung cancer patients [1]. As a result, cases of locoregional recurrence of cancer or the development of new cancers in previously treated areas have become more common. Patients with primary lung cancer are known to be at high risk of locoregional recurrence, second primary lung cancer, or metastasis to other parts of the lung [2]. Although re-treatment has been conducted for thoracic malignancies, including lung cancer, re-irradiation with curative intent has rarely been performed [3] due to the high risk of severe adverse events resulting from accumulated doses to organs such as the lung, esophagus, trachea, and proximal bronchial tree. Recently, however, with the introduction of intensity-modulated radiation therapy (IMRT) and stereotactic body radiation therapy, it has become possible to minimize the dose to surrounding organs while delivering a high dose to the target lesion. For these reasons, re-irradiation with curative intent has been increasingly performed even in cases with a history of thoracic irradiation [2,4]. However, in some cases, it is difficult to avoid irradiation of high-dose areas, making definitive radiotherapy impossible. We encountered a case where thoracic re-irradiation was successfully performed using IMRT. In this case, after definitive radiotherapy for esophageal cancer, definitive irradiation was performed again for newly developed lung cancer. As the radiation field in the treatment of esophageal cancer was close to the lung cancer lesion, delivering definitive radiation doses using three-dimensional conformal radiation therapy (3D-CRT) was expected to be challenging. Therefore, we used IMRT to enable the delivery of definitive doses. We created treatment plans for both IMRT and 3D-CRT and evaluated the doses. Here, we report on the feasibility of IMRT and review the relevant literature.

## Case presentation

**Figure 1:**
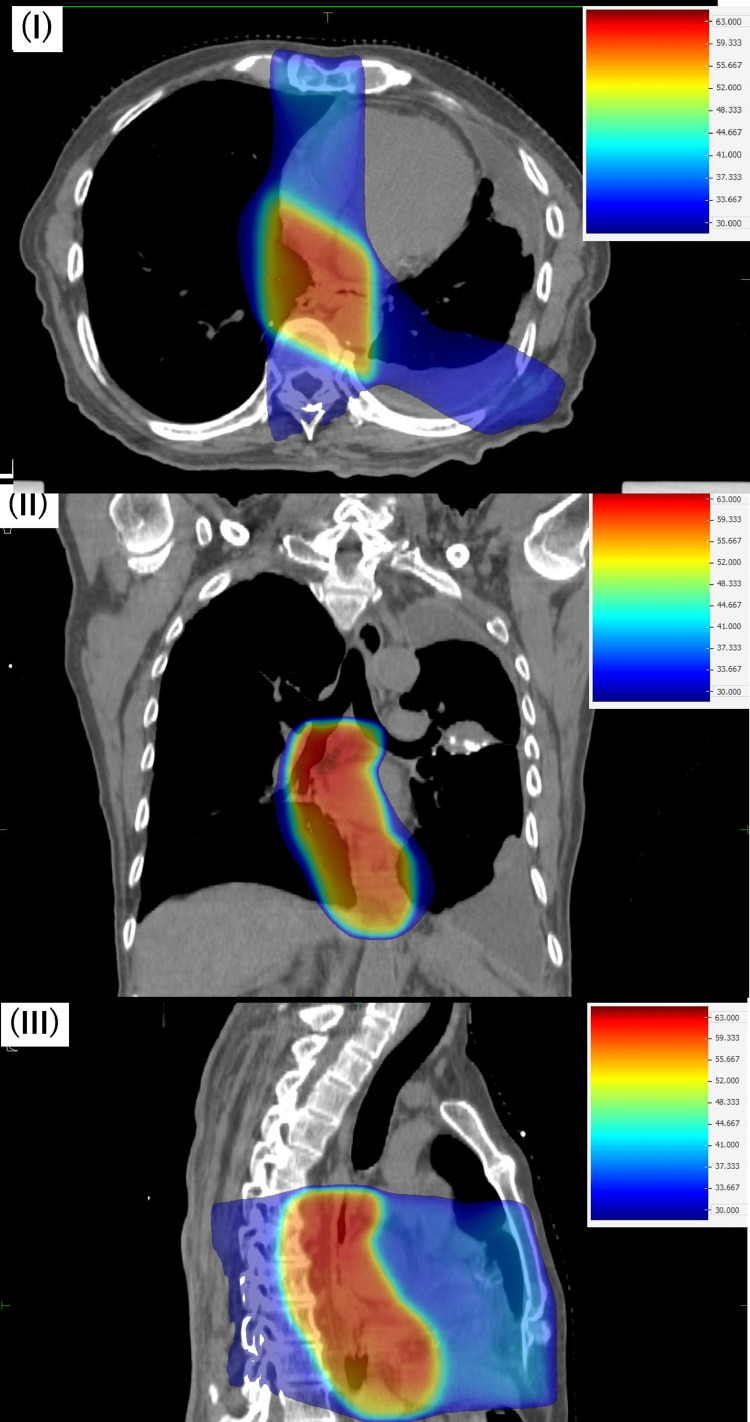
Treatment plan for esophageal cancer using three-dimensional conformal radiation therapy. I: dose distribution on an axial image; II: dose distribution on a coronal image; III: dose distribution on a sagittal image

The patient was a 74-year-old Japanese man. He had a history of esophageal cancer at the age of 69 and meningioma at the age of 72. Endoscopic submucosal dissection was performed for the esophageal cancer, which was located in the lower thoracic esophagus. Pathologically, it was diagnosed as squamous cell carcinoma, pT1bN0M0, pStage I (Union for International Cancer Control, eighth edition). As an additional treatment, postoperative radiation therapy using 3D-CRT was performed, delivering 60 Gy in 30 fractions. The dose distribution of this radiation therapy treatment plan is shown in Figure 1.

**Figure 2:**
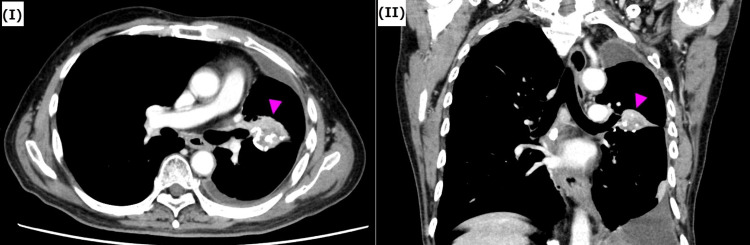
Contrast-enhanced chest CT. I: axial; II: coronal pre-treatment contrast-enhanced CT scans of the chest showing a 37×33×28 mm mass in left upper lobe lung around postoperative petz (arrowhead). Window center 30, window width 300.

For the meningioma, radiation therapy using the Cyberknife system was performed. The peripheral dose of 25 Gy was performed in three fractions. Additionally, the patient had a medical history of type 2 diabetes and hypertension. His social history included smoking 100 cigarettes per day for 45 years (from age 20 to 65) and drinking 3,500 ml of beer per day for 50 years (from age 20 to 70). At the age of 70 years, a nodule was identified in the upper lobe of the left lung. As a malignant tumor was suspected, S3+4+5 segmentectomy was performed. Pathologically, no malignancy was found. Three months after the surgery, a mass lesion appeared at the surgical site on computed tomography (CT); however, the mass was suspected to be a granuloma and was followed up. At the age of 73 years, follow-up CT revealed an increase in the size of the mass. A CT-guided percutaneous lung biopsy was performed on this mass lesion, and it was pathologically diagnosed as squamous cell lung carcinoma. A small amount of specimen was obtained from the biopsy which made it difficult to confirm further molecular analysis. In consultation with a pathologist, it was determined that the lesion was lung cancer and not a recurrence of esophageal cancer based on the differentiation and other factors. Contrast-enhanced CT revealed a mass lesion measuring 37×33×28 mm at the surgical site in the left lung (Figure 2).

**Figure 3:**
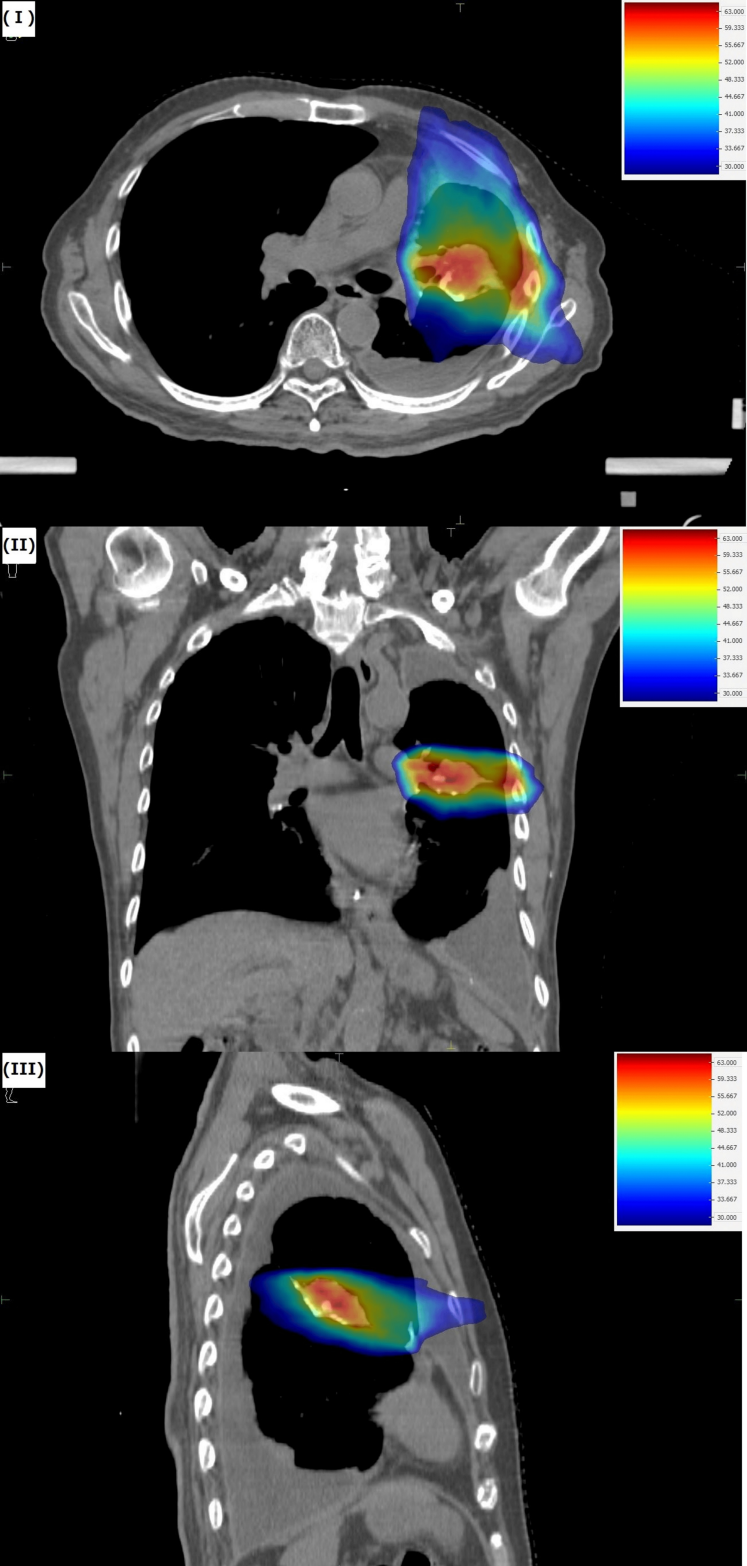
Treatment plan for lung cancer using intensity-modulated radiation therapy (treatment plan #2). I: dose distribution on an axial image; II: dose distribution on a coronal image; III: dose distribution on a sagittal image

There was no evidence of pleural invasion, main bronchus invasion, or separate tumor nodules. Additionally, there were no lymph node metastases or distant metastases. Based on these findings, we diagnosed the case as cT2aN0M0, cStage IB (Union for International Cancer Control, eighth edition). Current CT and endoscopic examination showed no signs of recurrence for the previously treated esophageal cancer. The lung showed pleural effusion. There was no obvious emphysematous lung or pneumonia. Meanwhile, spirometry results showed a vital capacity of 1.84 L, a percent vital capacity of 49.6%, a forced vital capacity of 1.56 L, and a percent predicted forced expiratory volume in 1s of 81.7%. Based on these results, we determined that the patient had restrictive ventilatory impairment. We formulated the following treatment strategy: since the esophageal cancer was recurrence-free, we determined that lung cancer was the prognostic factor and performed curative therapy for the lung cancer. Given the postoperative state of S3+4+5 segmentectomy and the restrictive ventilatory impairment, surgical resection, which is the primary option for non-small cell lung cancer according to the Japanese guidelines [5], was deemed high-risk due to the potential for further decline in lung function. After consulting with surgeons, we concluded that surgery was not feasible. Meanwhile, radiation therapy was considered as an option. However, there was a problem performing radiation therapy. The expected field in the current treatment was close to the irradiation field for the previous esophageal cancer treatment. We refer to the previous radiation treatment plan for esophageal cancer as treatment plan #1 (TP #1). If definitive radiation doses were delivered using 3D-CRT, it was anticipated that the lungs and esophagus would receive excess doses, potentially causing severe radiation damage. Therefore, we created a treatment plan (TP #2) using arc IMRT. The dose distribution is shown in Figure 3.

Upon evaluating TP #2, we determined that it was possible to reduce the doses to organs at risk that might otherwise be overexposed. Additionally, it could deliver a higher dose to the tumor, increasing the potential for cure. In TP #2, we targeted only the primary lesion, delivering 60 Gy in 20 fractions with six-megavolt X-rays. We used the Monaco treatment planning system version 5.51 (Elekta, Inc., Stockholm, Sweden) to create the treatment plan. To confirm the cumulative radiation dose, we calculated the total dose from TP #1 and TP #2 on the CT images used for the re-irradiation treatment plan. We referred to this as TP #1 + TP #2. Due to the difference in single-fractionation dose between TP #1 and TP #2, we calculated the equivalent dose in 2 Gy fractions (EQD2) using the following formula

**Figure 4:**
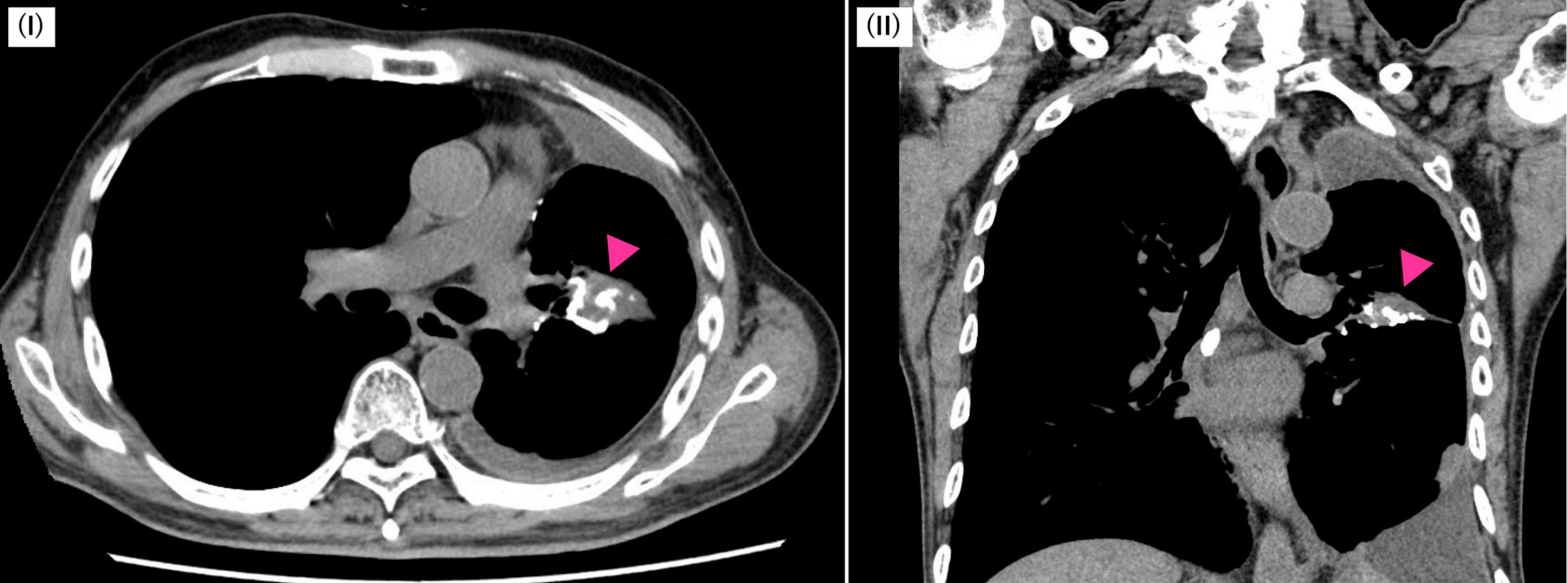
Plain chest CT. I: axial; II: coronal CT scan one month after radiotherapy shows a reduced mass of 30 x 21 x 17 mm in the left upper lobe lung around postoperative petz (arrowhead). Window center 30, window width 300.

EQD2=D×{d+(α/β)}/{2+(α/β)}

where the α/β values were 2.5 for the heart, 3 for the organs at risk, and 10 for the target lesion. We referred to the guidelines published by the American Radium Society (ARS) and the American College of Radiology (ACR) [6] to evaluate the doses to organs at risk. We determined that IMRT can minimize the doses to organs at risk while delivering a definitive dose to the cancer. We fully informed the patient and their family about the radiation therapy and obtained written consent from the patient. Subsequently, radiation therapy was completed without interruption. We used the Common Terminology Criteria for Adverse Events (CTCAE) version 5.0 to evaluate adverse events. No acute adverse events occurred. CT one month after radiotherapy showed a reduction in mass lesions (Figure 4).

As a late adverse event, grade 1 pleural effusion was observed, but radiation pneumonitis did not occur. There were no other severe adverse events. As of three years and six months after re-irradiation, there has been no recurrence of lung cancer. Eight years and six months have passed since the first esophageal cancer treatment, and no recurrence of esophageal cancer has been observed. Patients with primary lung cancer require careful follow-up because of the known high risk of local recurrence, second primary lung cancer, and metastasis to other parts of the lung. Late adverse events may occur after 10 or 20 years, and further follow-up is planned.

## Discussion

**Figure 5:**
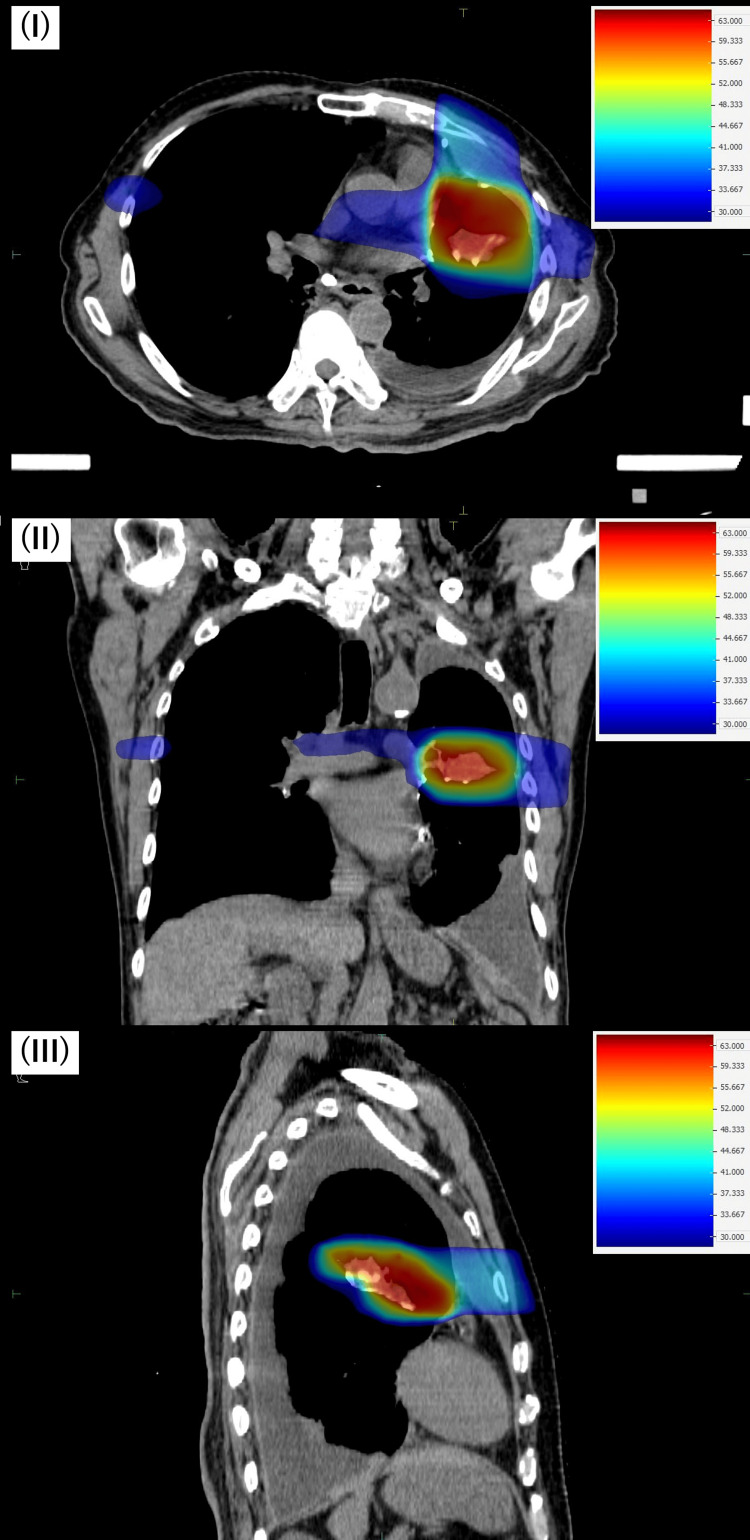
Treatment plan for lung cancer using three-dimensional conformal radiation therapy (treatment plan #3). I: dose distribution on an axial image; II: dose distribution on a coronal image; III: dose distribution on a sagittal image

We conducted thoracic re-irradiation using IMRT. By employing IMRT, we were able to minimize radiation doses to organs at risk while delivering definitive doses to the target lesion. To further evaluate the feasibility of IMRT, we had created an additional treatment plan using 3D-CRT, referred to as treatment plan #3 (TP #3). This plan targeted the same area with the same dose of 60 Gy in 20 fractions, similar to the IMRT plan (TP #2). The dose distribution for TP #3 is shown in Figure 5.

We confirmed the radiation field of TP #1 and created the treatment plan to ensure that no extremely high dose areas occur. We then compared TP #2 and TP #3. Comparisons of radiation doses to organs at risk and to the target lesion are shown in Tables 1, 2, respectively. In the 3D-CRT plan, irradiation was planned to avoid the high-dose area, and as a result, irradiation could be performed at an angle that avoided the spinal cord. IMRT was also planned to avoid the high-dose area but resulted in low-dose irradiation to the spinal cord. However, the dose to the spinal cord in the IMRT plan was below the tolerable dose.

**Table 1:** Comparison of radiation doses to organs at risk (i.e., the lung, main bronchus, spinal cord, esophagus, and heart) between treatment plan (TP) #2 and treatment plan (TP) #3. Dmean: mean dose; V 20 Gy: volume receiving 20 Gy; V 10 Gy: volume receiving 10 Gy; Dmax: dose maximum; V 50 Gy: volume receiving 50 Gy; V 60 Gy: volume receiving 60 Gy; V 40 Gy: volume receiving 40 Gy; D0.1cc: dose to 0.1 cc volume

Organs	Dose	TP #2	TP #3
Lung	Dmean(Gy)	6.48	8.61
	V 20 Gy(%)	8.76	15.44
	V 10 Gy(%)	23.79	24.63
Main bronchus	Dmax(Gy)	35.66	37.11
Spinal cord	Dmax(Gy)	9.29	0.82
Esophagus	Dmax(Gy)	18.10	38.01
	Dmean(Gy)	2.36	4.56
	V 50 Gy(%)	0.00	0.00
	V 60 Gy(%)	0.00	0.00
Heart	Dmean	7.34	7.73
	V 40 Gy	2.61	3.14
	D 0.1 cc	60.59	73.10

**Table 2:** Comparison of radiation doses to the GTV between treatment plan (TP) #2 and treatment plan (TP) #3. GTV: gross tumor volume; D 95%: minimum dose delivered to 95% of the GTV

GTV	TP #2	TP #3
Target Dmean(Gy)	59.39	60.50
Target D 95%(Gy)	53.15	47.45

Moreover, we evaluated the cumulative doses to organs at risk by combining TP #1 with TP #2 and TP #1 with TP #3. Several studies were used as references to assess the tolerance doses to organs at risk during thoracic re-irradiation. The guidelines published by the ARS and the ACR give tolerance doses for organs at risk during re-irradiation for non-small cell lung cancer [7]. We evaluated the doses of TP #1 + TP #2 and TP #1 + TP #3 based on these guidelines (Table 3). In TP#2 and TP#3 alone, the dose to the esophagus did not exceed 50 Gy. However, the dose to the esophagus exceeded 50 Gy in TP#1, resulting in cumulative doses exceeding V50 and V60. V60 was also higher in TP#1 + TP#2. This may be due to the fact that the low-dose range was wider than that of 3D-CRT because of the IMRT plan.

**Table 3:** Comparison of radiation doses to organs at risk (i.e., lung, main bronchus, spinal cord, esophagus, and heart) in treatment plan (TP) #1 + treatment plan (TP) #2 and TP #1 + TP #3 versus the dose constraints in the American Radium Society (ARS) and American College of Radiology (ACR) guidelines [6]. Dmean: mean dose; V 20 Gy: volume receiving 20 Gy; V 10 Gy: volume receiving 10 Gy; Dmax: dose maximum; V 50 Gy: volume receiving 50 Gy; V 60 Gy: volume receiving 60 Gy; V 40 Gy: volume receiving 40 Gy; D0.1cc: dose to 0.1 cc volume

Organs	Dose	TP #1+ TP #2	TP #1+TP #3	ARS and ACR guidelines
Lung	Dmean(Gy)	17.86	19.97	-
	V 20 Gy(%)	38.08	42.43	<40
	V 10 Gy(%)	53.24	56.03	-
Main bronchus	Dmax(Gy)	66.50	93.42	<110
Spinal cord	Dmax(Gy)	41.07	37.76	<57
Esophagus	Dmax(Gy)	67.76	94.09	<100-110
	Dmean(Gy)	41.20	43.43	-
	V 50 Gy(%)	63.32	64.84	-
	V 60 Gy(%)	22.17	19.42	<40
Heart	Dmean	31.84	32.24	-
	V 40 Gy(%)	35.76	36.06	<50
	D 0.1 cc	67.09	88.42	-

In TP #1 + TP #2, the dose to each organ at risk was below the ARS and ACR dose criteria. On the other hand, in TP #1 + TP #3, the dosimetric parameter of lung V20Gy did not meet the ARS dose criteria. Additionally, while the parameters of Dmax to the main bronchus, Dmax to the esophagus, and D0.1cc of the heart were below the ARS dose criteria, they were still relatively high.

We evaluated the tolerance doses and the risk of adverse events for the main bronchus, lung, esophagus, and heart based on several studies. The cumulative dose to the main bronchus in TP #1 + TP #3 resulted in a Dmax of 93.42 Gy, which is notably high. Rulach et al. [8] suggested a tolerance dose range of Dmax 80-105 Gy to the main bronchus during re-irradiation. Cannon et al. reported an increased risk of grade 4-5 adverse events, such as bronchocavitary fistula and massive hemoptysis, when the dose to the main bronchus exceeded 75 Gy in a series of treatments [9]. For the lung, the cumulative dose of V20Gy in TP #1 + TP #3 was high at 42.42%. V10Gy and Dmean were also relatively high in comparison with the IMRT plan. McAvoy et al. reported that the cumulative doses to the lung (V10Gy, V20Gy, Dmean) are correlated with the occurrence of radiation pneumonitis during re-irradiation [10]. For the esophagus, the cumulative dose of Dmax in TP #1 + TP #3 was also high at 94.09 Gy. Yang et al. found that in cases of esophageal re-irradiation, patients who experienced severe adverse events had higher cumulative doses of Dmax and V50Gy to the esophagus [11]. They also stated that in cases using 3D-CRT, it is difficult to adjust the irradiation dose to organs at risk, leading to a higher frequency of adverse events involving the esophagus. Xu et al. reported cases of esophageal re-irradiation where doses between 34-46 Gy resulted in esophageal fistulas or death due to major bleeding [12]. For the heart, the cumulative dose of D0.1cc in TP #1+TP #3 was high at 88.42 Gy. Paradis et al. set the tolerance dose of D0.1cc < 70-105 Gy for the heart during re-irradiation [13]. Taken together, these studies indicate that high doses to organs at risk during re-irradiation can significantly increase the risk of severe adverse events. Our results show that the IMRT plan (TP #1 + TP #2) reduced doses to organs at risk in comparison with TP #1 + TP #3. This might have sufficiently reduced the likelihood of severe adverse events such as bronchial hemorrhage, esophageal hemorrhage, bronchial fistula, radiation pneumonitis, esophagus fistula, and pericarditis. Our patient had no severe late adverse events and has remained recurrence-free for three years and six months of follow-up. Proton therapy can be used for re-irradiation for thoracic cancers. Proton therapy has the potential to reduce the area of relatively low doses using Bragg peaks. Thus, patients with low pulmonary function or underlying pulmonary disease may benefit from this approach. Yang et al. state that proton therapy may be more beneficial when compared to radiation therapy using Proton therapy and X-ray beams [11]. Since there were no facilities nearby, including our hospital, that could provide proton therapy, an X-ray beam was used in our case. It is possible that proton therapy will be used more widely in Japan in the future for re-irradiation of thoracic cancer.

Our report has several limitations. First, the patient underwent surgery for lung cancer after irradiation to the esophagus. The surgery involved a left S3+4+5 segmentectomy, resulting in a reduction of lung volume. This anatomical change before and after surgery may have affected the accurate evaluation of the cumulative dose in the treatment plan. Second, few prospective studies have investigated detailed tolerance doses during re-irradiation. Further accumulation of cases is required to determine more accurate tolerance doses. Lastly, the follow-up period was relatively short, at three years and six months. Since late adverse events can occur 10 or 20 years later, we plan to conduct further follow-up in the future. And there is a limitation to IMRT. The use of IMRT may increase the low-dose range. This may increase the low-dose range of organs at risk (OARs), such as the lungs. Although there are limitations as described above, we have demonstrated that employing IMRT can enable safe thoracic re-irradiation without causing severe adverse events. And with the advent of various technologies, such as AI technology-based contouring and treatment planning functions, IMRT is expected to continue to advance. With these advances in radiation therapy technology, more precise treatment will be possible in the future, even in cases that were previously considered difficult.

## Conclusions

We performed thoracic re-irradiation using IMRT. In this case, a definitive dose with 3D-CRT might have overexposed the lung and esophagus. However, using IMRT, we safely delivered a definitive dose. There have been no serious late adverse events, and there have been no recurrences even now, three years and six months later.
